# A Review of the Complex Relationship between Irritable Bowel Syndrome and Infertility

**DOI:** 10.3390/medicina56110592

**Published:** 2020-11-06

**Authors:** Carmen Anton, Alin Ciobica, Bogdan Doroftei, Radu Maftei, Ciprian Ilea, Natalia Darii Plopa, Maria Bolota, Emil Anton

**Affiliations:** 1 Department of Gastroenterology, Faculty of Medicine, “Gr. T. Popa” University of Medicine and Pharmacy, 6th University Street, 700490 Iasi, Romania; carmenro2008@yahoo.com; 2 Department of Biology, Faculty of Biology, “Alexandru Ioan Cuza” University of Iasi, Carol I Avenue, 20A, 700490 Iasi, Romania; 3 Department Center of Biomedical Research, Romanian Academy, Iasi Branch, Nr. 8, Carol I Avenue, No. 8, 700490 Iasi, Romania; 4 Department of Biology, Academy of Romanian Scientists, Splaiul Independentei Nr. 54, Sector 5, 050094 Bucuresti, Romania; 5 Department of Obstetrics and Gynecology, Faculty of Medicine, “Gr. T. Popa” University of Medicine and Pharmacy, 6th University Street, 700490 Iasi, Romania; bogdan.doroftei@umfiasi.ro (B.D.); cilea1979@yahoo.com (C.I.); emil.anton@yahoo.com (E.A.); 6 Clinical Department, Origyn Fertility Center, Palace Street, No 3C, 700032 Iasi, Romania; dr.radu.maftei@gmail.com; 7 Department of of Obstetrics and Gynecology, Grand Hôpital De Charleroi, Avenue du Centenaries 73, 6061 Charleroi, Belgium; plopa_nati@yahoo.com; 8 Department of Obstetrics and Gynecology, Spitalul Clinic de Obstetrică și Ginecologie Cuza Vodă, 700032 Iasi, Romania; bolota882003@yahoo.com

**Keywords:** IBS, infertility, oxidative stress, gynecology, gastrointestinal, ROS

## Abstract

Irritable bowel syndrome (IBS) is a gastrointestinal disease that negatively affects up to 20% of the population. Infertility is defined as a disorder of the reproductive system described by lack of success in achieving pregnancy after more than a year of regular unprotected sexual intercourse. The main purpose of our review was to analyze the available literature regarding the IBS-infertility connection. Another secondary purpose of the present paper was to find out if oxidative stress may be the missing puzzle that may explain this possible correlation. After analyzing the available literature we concluded that oxidative stress is a plausible mediator of the connection between both female and male fertility and IBS. However, the data lacks in direct evidence to confirm this hypothesis. Nevertheless, it is recommended that certain levels of oxidative stress should not be exceeded in order to decrease IBS symptoms and increase the odds of conception given that generation of reactive oxygen species (ROS) is an aftermath of metabolically active cells. Therefore, reducing the oxidative stress by living a healthier lifestyle with a balanced diet, rich in micronutrients, limited in caffeine and alcohol, avoiding smoking and maintaining a normal body mass index with regular physical exercise may promote fertility and help diminishing IBS symptomatology. Studies with measurements of biological samples are needed in order to assess the complex relationship between oxidative stress, IBS and infertility.

## 1. Introduction

Irritable bowel syndrome (IBS) is a frequent gastrointestinal disease that negatively affects up to 20% of the population worldwide [1,2]. IBS has a clear gender difference, as it affects more women than men, studies showing that women are three times more likely to have IBS compared to men [3]. The correlations between sex hormones, especially progesterone, and gastrointestinal function have been proposed as an explanation on why women are affected by IBS to a larger extent compared to men [4,5].

Another specific characteristic of this disease is that IBS is more common diagnosed in individuals who are younger than 50 years old [6]. The main symptoms of IBS include chronic abdominal pain and/or discomfort, diarrhea, constipation and bloating. These symptoms are present in some patients daily, while other patients have these symptoms intermittent, at intervals of weeks or rarely, even months [7]. The importance of studying IBS comes from the fact that this disease considerably decreases the quality of life. Although the etiopathogenic causes of IBS are considered to be related to diet, gut microbiota, low levels of inflammation and disruptions in the function of enterochromaffin cell accompanied by genetic factors, the precise cause of IBS remains unclear [8,9].

In the literature, infertility is defined as a disorder of the reproductive system described by lack of success in achieving pregnancy after more than a year of regular unprotected sexual intercourse [10]. Therefore, the main purpose of our review was to analyze the available literature regarding the IBS-infertility connection. Another secondary purpose of the present paper was to find out if oxidative stress may be the missing puzzle that may explain this possible correlation.

## 2. Methodology

The inquired databases (from inception till July 2020) were: Medline, Embase, DARE databases and the Cochrane Database of Systematic Reviews (CDSR). The keywords used in the search strategy were: “infertility IBS connection”, “infertility oxidative stress correlation”, “IBS oxidative stress relationship”, “infertility reactive species of oxygen”, “IBS reactive species of oxygen”, “antioxidants IBS”, “infertility antioxidants”, “IBS superoxide dismutase”, “Infertility superoxide dismutase”, “IBS catalase”, “infertility catalase” “infertility glutathione peroxidase” and “IBS glutathione peroxidase”.

## 3. Irritable Bowel Syndrome and Infertility

Although the relationship between fertility and IBS has been examined in the literature, relatively little is known regarding the reproductive disorders faced by patients who suffer from IBS.

Therefore, the available studies that examine male infertility in IBS patients reported results showing that infertility is more prevalent in individual with IBS compared to the general population [11]. Furthermore, one case control study [12] concluded that the number of children born to individuals with IBS is significantly lower when compared to patients suffering from other gastrointestinal disorders, such as ulcerative colitis, and the general population. Furthermore, the reported results from the same study showed no statistically significant difference in the number of children that individuals with ulcerative colitis have and the general population [8]. Moreover, the significant differences observed between patients with IBS and controls could not be explained by the fecundability or the frequency of the sexual intercourse as these two measured variables did not differ significantly in the two groups [13,14].

Another interesting explanation of these results was proposed by Heetun et al [15] who suggested that the decision to limit family size may be explained by fear of passing IBS predisposition genes rather than a physical effect of the disease itself. This hypothesis is backed by another study which showed that IBS suffering individuals presented a reduction in fertility up to 50% with no significant difference measured in the reproductive capacity [16].

However, even if IBS itself does not seem to physically influence fertility, the medications which are commonly prescribed to treat this disorder could definitely affect one individual’s ability to procreate. An unwanted surgical procedure or malnutrition resulting from IBS may provoke various sexual dysfunctions that may finally lead to infertility [17].

Therefore, patients suffering from IBS should consider changing their prescribed pharmaceutical treatment. For example, individuals who have a prescription for sulfasalazine, should substitute this drug with a different 5-ASA. It is recommended that this change in medication to be made at least 4 months before any attempt to conceive [18]. Furthermore, in order to restore fertility, patients with IBS who are on mesalazine are also advised to discontinue the treatment [19]. However, to avoid any potential escalation of IBS symptoms, corticosteroids are advised to be used to control the active disorder [20]. In addition, although some authors recommend the interruption of methotrexate (MTX) treatment 4 months prior to attempting to conceive, there is simply not enough evidence to support this guidance. The risk of an acute increase in IBS symptoms as a result of MTX discontinuation might eclipse any yet to be proven benefits regarding fertility [21]. The interruption of the treatment is recommended only if any erectile dysfunctions are present. Furthermore, there is also not enough data to endorse interruption of thiopurines and anti-TNF (tumor necrosis factor) agents such as IFX (infliximab) [22]. Moreover, to individuals who intend to undergo proctocolectomy sperm banking prior to the procedure should be offered because postoperative anejaculation, although a rare side effect, is a possible permanent complication [23].

Beside the medications, other possible explanations on the observed high levels of infertility in IBS suffering men are active inflammation, alcohol consumption, smoking habits, use of over the counter medications and unhealthy nutritional intake [24].

Therefore, before any treatment for infertility should be prescribed it is important to try control any possible variables that may worsen IBS symptoms. For example, if the individual has unhealthy nutritional habits, optimizing their food intake, with proper macro and micronutrients partitioning, is recommended [25]. Smoking cessation should strongly be advocated if the patient has a smoking habit.

In the majority of the available studies the molecular mechanism behind the connection between IBS and infertility is related to gonadotropin-releasing hormone (GnRH) [26]. This hormone is the hypothalamic hormone in the sex hormone axis. GnRH is known to stimulate the release of estrogen and progesterone [26].

Furthermore, GnRH neurons are demonstrated in the literature to control fertility in all mammalian species [27]. In addition, studies show that humans who present a dysfunction connected to the GnRH neurons are also infertile [28]. Moreover, animal models also proved that mice with defective GnRH biosynthesis are also sterile [29].

Another explanation on the correlation between infertility and IBS is considered in the literature to be connected to the prescribed treatment of infertility, the in vitro fertilization intervention (IVF). The problem occurs when IVF is prescribed repeatedly to women who have problems conceiving, the same population, as before mentioned, which is most likely to be affected by IBS [26]. Although this possible correlation has not been extensively studied, the limited available data shows that IBS symptoms do occur after IVF [30].

However, perhaps the most important variable that may explain the IBS–infertility relationship is stress related. Reactive oxygen species (ROS) are created by cells in some physiological and pathological situations. The main ROS are O_2_^−^ (superoxide), OH^−^ (hydroxyl), H_2_O_2_ (hydrogen peroxide), ^1^O_2_ (singlet oxygen) and NO (nitric oxide). These ROS are known to respond to all macromolecules, lipids, proteins and carbohydrates. However, they particularly react with polyunsaturated fatty acids on the membrane of the cell. Cell injury and ultimately cell death occurs after the initial reaction with ROS and after the following chain reaction is initiated [29]. However, ROS-started oxidative stress can be controlled by the antioxidant defense system. The antioxidant defense mechanism includes enzymatic and nonenzymatic antioxidants. The main enzymes that have antioxidant properties are superoxide dismutase (SOD), catalase (CAT) and glutathione peroxidase. Regarding the nonenzymatic antioxidants, these are represented by vitamin E, vitamin C and flavonoids [31].

Thus, oxidative stress results when the generation of ROS surpasses the ranging capacity of antioxidants as a result of two possible situations: high levels of ROS and/or deficient intake or elevated usage of antioxidants. The majority of ROS are created as a result of the mitochondrial respiratory chain. However, ROS can also be created by various exogenous factors. These exogenous factors include alcohol consumption, tobacco smoking and various environmental pollutants [32]. As it can be seen in Table 1, all infertility factors in patients who are suffering from IBS from our review are also linked to oxidative stress. Therefore, we decided to explore the available literature to analyze both oxidative stress- infertility and oxidative stress-IBS relationships.

## 4. Oxidative Stress and Infertility

Accordingly, the next correlation we reviewed in the present paper is the oxidative stress- infertility one. In addition, since infertility in men and women are two separate disorders with different pathogenesis, we created two separated subsections.

### 4.1. Males

In male subjects, specific studies demonstrated that a high level of oxidative stress leads to decreased sperm motility, sperm number and sperm–oocyte fusion [38]. The exact mechanism behind the negative influence of oxidative stress on male infertility is connected to spermatozoa. A human spermatozoon is a cell type that has the capacity to generate ROS when incubated under aerobic conditions. The production of ROS by sperm is considered a common physiologic process [39]. The problem occurs when an imbalance between ROS generation and scavenging activity develops. This imbalance is associated in the literature with male infertility because it is detrimental to the sperm [40,41].

Therefore, it is vital to detect the sources from where ROS is produced in order to understand the impact of high levels of ROS on the spermatozoa, especially in those phases that are vital for fertilization.

Thus, human spermatozoa have the capacity to produce ROS by starting the peroxidation of the unsaturated fatty acids that are found in the sperm plasma membrane [42]. This process plays an important role in the etiology of male infertility.

These molecules have a relatively short half-life and limited diffusion, which is accordant with their physiologic function in acrosome reaction and hyper activation. Therefore, a major cause of defective sperm function in cases of male infertility may be the intrinsic reactivity of the before mentioned metabolites in peroxidative damage. This may be induced by high levels of ROS production, particularly H2O2 and the superoxide anion [43].

In addition, the two main sources of ROS production in the reproductive system of the men are the spermatozoa and infiltrating leukocytes [44]. Although both spermatozoa and leukocytes have the capacity to produce ROS, leukocytes are known to produce much more elevated levels [45]. It is important to mention that the clinical significance of leukocyte production of ROS in semen is controversial. Seminal plasma contains enzymes that scavenge ROS, such as catalase and superoxide dismutase, offer protection against ROS damage. Therefore, a number of defense mechanisms can be used to reduce the levels of oxidative stress caused by excessive ROS production [46].

Therefore, developing effective therapies to overcome excessive ROS production is important in order to help reduce male infertility, given that ROS production can have both positive and negative effects on the spermatozoa and equilibrium between the levels of ROS produced and the levels of ROS scavenged will decide if a given sperm function will be promoted or endangered [47]. 

Thus, precise measurement of ROS production levels is vital, because it will help clinicians identify patients that may or may not respond to specific therapeutic plans, and perhaps more important it will help elucidate the fertility status of an individual. 

However, the possible benefits of various antioxidant supplements for fertility purposes must be cautiously investigated, until proper clinical trials validate them. From the current available data it appears that there is still no single adjuvant treatment that is able to enhance the fertilizing ability of sperm in infertile males [48]. A more feasible approach would be a combination of various medications that are not toxic at exact dosages used.

### 4.2. Females

On the other hand, animal models, in-vitro data and human studies demonstrated that high levels of oxidative stress may negatively affect female fertility [49]. However, the exact mechanism behind this observed correlation has not been yet discovered in subjects who are trying to conceive.

It has been demonstrated that high levels of oxidative stress negatively influence both natural and assisted fertility [50]. Various oxidative stress biomarkers have been discovered in different locations in the female reproductive tract. These finding are suggesting that oxidative stress biomarkers are playing important roles in various physiological functions [51]. However, a number of studies have suggested that ROS production may be a causative factor of infertility. This causal link may explain various types of infertility, such as peritoneal factor, tubal factor, endometriosis and unexplained infertility [51]. However, the scientific cause of unexplained infertility remains unclear, but oxidative stress may be proven to play an important role in its pathophysiology.

Even the role of oxidative stress in other types of infertility is not completely discovered. Many studies have measured the potential role of various markers of the oxidative stress in tubal factor infertility, endometriosis and peritoneal factor infertility [52]. The results reported by these studies showed that the tubal and peritoneal microenvironments impact fertilization and early embryonic growth. If high concentrations of ROS are found in these environments, they might produce negative effects both in the Fallopian tube and the peritoneal cavity, on the spermatozoa, oocytes, sperm-oocyte interaction and fetus [53]. Furthermore, activated macrophages have been found to play an important role in the process of pathogenesis of endometriosis. In the peritoneal environment affected by endometriosis, these macrophages are capable of producing high levels of ROS.

## 5. IBS and Oxidative Stress

Once we established that a connection may in fact exist between oxidative stress and infertility, we proceed in analyzing the available data regarding the oxidative stress influence on IBS symptoms. The potential role of oxidative stress in IBS has been demonstrated in clinical and experimental studies [54].

Nevertheless, in order to measure the significance of the possible correlation between IBS and oxidative stress, the non-enzymatic antioxidant and antioxidant enzymes plus the nitric oxide and lipid peroxidation product activities must be evaluated (Table 2). Our analysis of the literature found only one study in which plasma xanthine oxidase activity was measured in patients suffering from IBS [48]. Furthermore, the results reported by the authors of this study showed that patients with IBS had higher levels of the enzyme activity in comparison with the control group [55]. Xanthine oxidase is known for decreasing molecular oxygen to hydrogen peroxide and superoxide anion radical. This process is possible only in the presence of its substrate hypoxanthine or xanthine. Furthermore, xanthine can further react to create the more reactive hydroxyl radical, which is a reactive oxygen species. This xanthine oxidase derived reactive oxygen species have been found to be one of the critical factors in mechanisms of various pathophysiologies, including IBS [55]. IBS is a digestive system mucosal disorder represented by altered humoral and cellular immunities [56]. Therefore, studies that examined the relationship between IBS and various oxidative stress markers showed that patients with IBS present reduced levels of mucosal-soluble mediators and adhesion molecules [56] and elevated inflammatory cytokine levels [57]. Furthermore, in another study on the IBS-oxidative stress correlation, the results reported by the authors showed that patients with IBS had decreased extracellular matrix components and matrix receptors [58].

Although the exact molecular mechanism behind oxidative stress’ role on the symptomology of IBS remain unclear, it has been proposed the idea that reactive oxygen species derived from xanthine oxidase may contribute to elevated expressions of mRNA for interleukin 1 beta (IL-b) and tumor necrosis factor-a (TNF-a) [59]. Both increased expressions of mRNA for interleukin beta (IL-b) and TNF-a are also found in patients who are suffering from IBS [59].

Another proposed molecular mechanism that might explain oxidative stress’ connection with IBS refers to the role of inflammation as a causative element of altered intestinal activity. Therefore, inflammatory factors such as IL-1 and H_2_O_2_ have also been demonstrated to be correlated with abnormal sigmoid motor function in other gastro-intestinal disorders such as ulcerative colitis [60]. In addition, IL-1 it is well known to be generated by the release of several ROS, including H_2_O_2_ [61]. Moreover, ROS have an important role in the activation of intracellular signalling pathways such as those affected by the IL-1 signal [62]. Therefore, ROS, including H_2_O_2_, may negatively influence the activity of various crucial transcription factors and, consequently, the matrix gene expression of fibronectin. We advocate that a similar molecular process is present in the intestines of individuals who are suffering from IBS.

Therefore, our review indicates that lipid peroxidation and alterations in the oxidant-antioxidant enzymatic structure may be implicated in the pathogenesis of IBS. Furthermore, elevated levels of lipid peroxidation observed in IBS patients may be explained by an escalation of xanthine oxidase activity and a reduction of the activity of the antioxidant enzymes. In addition, we believe that our review brings important evidence suggesting that oxidative stress plays an important role in the pathophysiology of the irritable bowel syndrome. Moreover, we highlight the importance of future research for the treatment of this disease with the development of medication aiming to help these complex pathways.

In addition, maintaining a healthy body weight is another simple and effective method to reduce oxidative stress and therefore decrease IBS symptoms and increase pregnancy odds. The literature clearly shows that female obesity negatively influences fertility by altering various hormone patterns and the menstrual cycle. For example, a study demonstrated that a high BMI (body mass index) is correlated with low levels of antioxidant markers and high levels of oxidative stress. Furthermore, protein oxidation in the placenta was measured and the results reported by the authors demonstrated that placental oxidative stress increased with the body weight of the mother [63]. Although the effects of specific diets, meal plans and the possible role of oxidative stress is yet to be studied, given that obesity affects up to 75% of women with polycystic ovarian syndrome (PCOS) [33], many researches have demonstrated that losing weight increases fertility among women in general and especially in those suffering from PCOS [34,35,36].

## 6. Conclusions

Oxidative stress is a plausible moderator of the connection between both female and male infertility and IBS, but the available data from the literature lacks in direct evidence to confirm this hypothesis. It is recommended that certain levels of oxidative stress should not be exceeded in order to decrease IBS symptoms and increase the odds of conception given that generation of ROS is an aftermath of metabolically active cells. Furthermore, reducing the oxidative stress by living a healthier lifestyle with a healthier diet, rich in micronutrients, limited in caffeine and alcohol, avoiding smoking and maintaining a normal body mass index with regular physical exercise may also promote fertility and diminish IBS symptomatology. Nonetheless, prospective studies with strict measurements of various biological samples are needed in order to assess the complex relationship between oxidative stress, IBS and infertility.

## Figures and Tables

**Table 1:** Possible causes of infertility in irritable bowel syndrome (IBS) patients.

Factors	Studies
Obesity	Roberts et al [33], Ehrmann [34], Stamets et al. [35], Moran et al. [36]
IBS Medication	Sands et al. [19], Shin et al. [20]
Smoking	Feagins et al [18]
Alcohol consumption	Feagins et al [18]
Poor nutritional habits	El Tawil 2003 [37]
Psychological factors	Heetun et al. [15], Tavernier et al. [16]

**Table 2:** Relevant studies for each of the investigated correlation.

Investigated Relationship	Relevant Studies
Irritable bowel syndrome-Infertility	Rossato et al. [11], Moody et al. [12] Narendranathan et al. [13], Lee et al. [17]
Oxidative stress-Infertility	Sharma et al. [39], Aitken et al. [43] Agarwal et al. [51]
Oxidative stress-Infertility	Mate et al. [64]
